# Anlotinib Suppresses Oral Squamous Cell Carcinoma Growth and Metastasis by Targeting the RAS Protein to Inhibit the PI3K/Akt Signalling Pathway

**DOI:** 10.1155/2021/5228713

**Published:** 2021-12-08

**Authors:** Yunfei Lu, Jing Lin, Meng Duan, Ying Rui, Hao Zheng, Liting Zhu, Xuanyu Zhu, Jingchen Wei

**Affiliations:** ^1^College of Pharmacy, Guilin Medical University, Guilin 541199, China; ^2^Department of Medical Oncology, The Affiliated Hospital of Guilin Medical University, Guilin 541001, China; ^3^Research Lab of Translational Medicine, Heng Yang Medical University, University of South China, Hengyang 421001, China; ^4^Guangxi Liver Injury and Repair Molecular Medicine Collaborative Innovation Center, Guilin Medical University, Guilin 541199, China; ^5^Guangxi Bagui Scholars Program, Guilin Medical University, Guilin 541199, China

## Abstract

Oral squamous cell carcinoma (OSCC) is a malignant tumour originating from the mucosal lining of the oral cavity. Its characteristics include hidden onset, high recurrence, and distant metastasis after operation. At present, clinical treatment usually includes surgery, chemotherapy, radiotherapy, or the joint use of these modalities. Unfortunately, multidrug resistant is one of the important obstacles that causes cancer chemotherapy failure. Anlotinib, which has recently been proven to have good antitumour effects, is a novel multitargeted tyrosine kinase inhibitor. However, there are few studies of the anlotinib-associated mechanism in OSCC and its underlying molecular mechanism. In our study, in vitro models of human oral squamous cell carcinoma HSC-3 cells were used to determine the efficacy of anlotinib. On the one hand, we showed that anlotinib treatment significantly reduced the viability and proliferation of HSC-3 cells and decreased cell migration by inhibiting the activation of the Akt phosphorylation pathway. On the other side, anlotinib inhibited PI3K/Akt/Bad phosphorylation and promoted apoptosis of HSC-3 cells by activating RAS protein expression. In brief, these results indicated that anlotinib had prominent antitumour activity in OSCC, mainly by inhibiting the PI3K/Akt phosphorylation pathway. This work provides evidences and a basic principle for using anlotinib to treat patients with OSCC for clinical research.

## 1. Introduction

Oral squamous cell carcinoma (OSCC) is the most common malignant tumour of the head and neck [1] and is estimated to cause 200000 new tumours worldwide each year [2]. The current treatment methods for OSCC are mainly surgical treatments combined with postoperative radiotherapy and chemotherapy. However, due to the strong migration and invasion abilities of OSCC, patients easily relapse and have poor prognoses, with a 5-year survival rate of only 55% [3]. For OSCC that cannot be treated with surgical therapy, combination therapy such as cisplatin has now become the first-line option for OSCC patients. Other first-line treatment drugs are mainly used, but their clinical effects are poor [4]. Therefore, it is urgent for us to achieve a better understanding of the molecular mechanism of OSCC. It can help us better find new ways of treatment contribute to the further treatment of tumours and build up the survival probability of patients.

Anlotinib is a novel small-molecule multitarget tyrosine kinase receptor inhibitor that is popular due to its anticancer properties and new side effects [5, 6]. In a recent study, anlotinib mainly inhibited angiogenesis and tissue tumour cell migration by inhibiting c-Kit, VEGFR2, VEGFR3, FGFR1-4, PDGFR *α* and *β*, and other targets, thus inhibiting tumour [7, 8]. Moreover, according to the results of comprehensive clinical trials, compared with apatinib and imatinib, anlotinib has a better targeted therapeutic effect, better general therapeutic effect, and lower side effects. Compared with other first-line antitumour drugs, anlotinib has fewer side effects and a higher five-year survival rate [9]. Consequently, it is of great consequence to understand the various molecular mechanisms of anlotinib in the treatment of tumours to provide better treatment.

As a considerable member of the GTP-enzyme gene family, the Ras gene regulates cell proliferation, mutation, and apoptosis by encoding the RAS protein H-ras regulates downstream silk/threonine protein kinase B (Akt) by regulating the p110 subunit of phosphoinositol-3-kinase (PI3K). It regulates of protein synthesis and promotes the secretion of vascular endothelial growth factor to inhibit cell apoptosis [10]. In addition, when Akt is phosphorylated, the proapoptotic protein Bad can also be phosphorylated by Akt. Phosphorylated Bad binds to the 14-3-3 protein subtype to form a complex that remains in the cytoplasm and plays a role in inhibiting apoptosis. Therefore, the Bad protein is directly correlated with the PI3K/Akt pathway and the apoptotic mechanism [11]. Unfortunately, there has been no study on the role of tyrosine kinase inhibitors in RAS protein promotion of apoptosis. Here, we explored the pharmacological effect of anlotinib on the RAS protein and its possible molecular mechanisms.

In this investigation, we aimed to evaluate the potential functional roles of anlotinib in OSCC in vitro and explore the molecular mechanism comprehend in this process. This work provides proof-of-concept for the clinical evaluation of anlotinib in the treatment of OSCC.

## 2. Materials and Methods

### 2.1. Chemicals and Agents

Anlotinib (dihydrochloride form; purity > 99%) was procured from Jiangsu Chia-tai Tianqing Pharmaceutical Co., Ltd. (Nanjing, China). MTT was purchased from Solarbio (Cat. No. M8180 Shanghai, China). Antibodies toward RAS (Cat. # AF0247), PI3K (Cat. # AF6241), Akt (Cat. # AF6261), p-Akt (Cat. # AF0016), Bad (Cat. # AF6471), MMP-2(Cat. # AF0577), MMP-9 (Cat. # AF5228), and *β*-actin (Cat. # AF7018-BP) were purchased from Affinity Antibody (Shanghai, China).

### 2.2. Cell Culture

Human oral squamous cell carcinoma (HSC-3) cells were procured from the Chinese Academy of Sciences, Cell Bank of the China (Shanghai, China). All cells were cultured in RPMI-1640 medium supplemented with 10% foetal bovine serum, 100 U/ml penicillin, and 100 mg/ml streptomycin at 37°C with 5% CO_2_.

### 2.3. MTT Assay

The MTT assay was enforced as formerly described [12]. In short, the cells were collected, and the cell suspension concentration was adjusted in a petri dish with 10% 1640 complete medium. The cells were seeded into 96-well plates at 6000 cells per well. Edge holes were filled with sterile PBS, and the cells were cultured in a 37°C and 5% CO_2_ incubator to the logarithmic growth phase. They were uncoupled into a blank group, a negative group, and an administration group. In the negative group, drug-free medium was added to fill the volume, and the cells were incubated in a constant temperature incubator (37°C, 5% CO_2_) for 24 h, 48 h, and 72 h, respectively. MTT then added. In the back of 4 h of reaction, the supernatant was discarded; the absorbance of the 96-well plate with DMSO was measured at 490 nm with a microplate reader.

### 2.4. Colony Forming Assay

Colony forming assays were performed as previously described [12]. Briefly, the collected and cultured cells were diluted with 10% 1640 complete medium, and approximately 300 cells per well were added to 6-well plates. The cells were cultured in a constant-temperature incubator at 37°C and 5% CO_2_ until the cells adhered to the wall. Discard the complete media without drugs, and add the complete media with drug concentrations of 1 mol/L, 2 mol/L and 4 mol/L for culture, and the control wells were set. The culture medium with the corresponding drug concentration was changed every two days, and the culture medium was discarded after 10 days of culture. Five hundred microlitres of fixative was added to each well for 30 min, after which the fixative was discarded. Then, 500 *μ*L crystal violet dye solution was added to each well for 30 min. The dye was washed away, and the bacterial formation rate was calculated after drying.

### 2.5. Wound Healing Assay

The wound healing assay was performed as previously described [12]. In short, HSC-3 cells in the logarithmic growth phase were selected and inoculated into 6-well plates after adjusting the cell density to 5 × 10^5^ cells/well. After the cells grew and the monolayer covered the bottom of the well, three parallel lines were drawn under each well to form scratches. After removing the original medium, each well was washed with PBS three times. Serum-free medium containing drugs was added, and serum-free medium without drugs was set as the control group. Cell migration was observed at 0 h and 24 h, respectively.

### 2.6. Immunofluorescence

Immunofluorescence was implemented as described in a previous article [13]. Logarithmic growth-phase HSC-3 cells were seeded into 24-well plates with coverslips at 1 × 10^4^ cells/well. When the cells grew to 40%, the culture medium was removed, positive cells with drug concentrations of 2.5, 1.25, and 0.625 *μ*mol/L were added, and the control group was set up. After 24 hours of reaction, the cells were washed with PBS three times and then fixed with 4% polymethanol for 10 min at 4°C. The fixative was removed, and the cells were rinsed PBST three times. The cells were blocked with 5% BSA for 30 min and washed with PBST 5 times. p-Akt antibody (1 : 100) was added and incubated with the cells at 4°C for 8 h. The cells were washed five times with PBST and then incubated in a refrigerator at 4°C for 2 h in the dark with the secondary antibody (1 : 100). The cells were washed with PBST 5 times, 200 *μ*L DAPI was added for 10 min, and the cells were again washed with PBST 5 times. An appropriate amount of antifluorescence quenching agent was added to the sealing area, the glass slide was fixed with resin, and photos were taken under a fluorescence microscope.

### 2.7. Hoechst 33258 Staining

Hoechst 33258 staining was performed as described in a previous article [14]. Cells in the logarithmic growth phase were collected and added to 6-well plates after adjusting the concentrations. The 6-well plates were placed in a constant-temperature incubator at 37°C and 5% CO_2_ until cells reached the logarithmic growth phase. The medium was discarded, and medium containing drugs was added to the control group. After 72 h, the medium was discarded, and the cells were washed twice with PBS and fixed in 500 *μ*L for 10 min. Then, the cells were washed with PBS twice and stained with Hoechst 33258 dye (C1011 Beyotime, ShangHai) for 5 min. Then, the dye was washed away with PBS, and the antifluorescence quenching agent was added dropwise.

### 2.8. Western Blot Analysis

After treatment with anlotinib, total cellular proteins were obtained using RIPA cell lysis buffer, and the concentrations were determined using BCA. From each sample, 20 *μ*g of total proteins was separated by SDS-PAGE and further transferred onto nitrocellulose membranes. The membranes were blocked in blocking buffer (5% BSA in Tris-buffered saline with 0.1% Tween-20 (TBST)) for 2 h and then incubated with primary antibodies including rabbit anti-RAS (1 : 1000), rabbit anti-PI3K (1 : 1000), rabbit anti-p-Akt (1 : 1000), rabbit anti-Akt (1 : 1000), rabbit anti-Bad (1 : 1000), rabbit anti-MMP-2 (1 : 500), rabbit anti-MMP-9 (1 : 500), and rabbit anti-*β*-actin (1 : 1000) diluted with 5% BSA in 0.01 M TBST at 4°C overnight. The membranes were washed in TBST and further incubated with horseradish peroxidase- (HRP-) conjugated secondary antibody (1 : 10000) diluted in 0.01 M TBST for 1 h at room temperature [15].

### 2.9. Statistical Analysis

Data are expressed as the mean ± SD obtained from triplicate experiments. Statistical analysis was performed by one-way analysis of variance (ANOVA) using SPSS 25. Values obtained in the assays were considered statistically significant when *P* < 0.05.

## 3. Result

### 3.1. Anlotinib Inhibits Oral Squamous Cell Carcinoma HSC-3 Cells Cell Growth in a Dose-Dependent Manner

**Figure 1:**
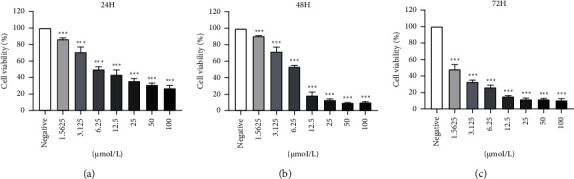
Anlotinib inhibits HSC-3 cell growth. MTT assay tested the cell proliferation was determined. Different doses of anlotinib (0, 1.5625, 3.125, 6.25, 12.5, 25, 50, and 100 *μ*M) inhibited the proliferation of HSC-3 cells after 72 h of treatment. The data are expressed as the mean ± S.E.M., *n* = 3 with available differences from the control designated by ^∗∗∗^*P* < 0.001: (a) 24 h; (b) 48 h; (c) 72 h.

Anlotinib has been approved as a single drug therapy for metastatic non-small-cell lung cancer or locally advanced in China. The recommended dose of anlotinib is 12 mg once a day for two consecutive weeks repeated once every three weeks until disease progression or unacceptable tolerance [8]. Thus, to explore the effectiveness of anlotinib on HSC-3 human oral squamous cell carcinoma, we first detected the half maximum inhibitory concentration (IC50) of anlotinib by MTT assay. The data showed that the IC50 values of HSC-3 at 24 h, 48 h, and 72 h were 11.7307 *μ*mol/L, 6.6207 *μ*mol/L, and 1.001 *μ*mol/L, respectively (Figure 1). Consequently, anlotinib significantly forborned the diffusion of HSC-3 cells in a dose-dependent mode in vitro.

### 3.2. Anlotinib Suppressed Oral Squamous Cell Carcinoma HSC-3 Cell Proliferation

**Figure 2:**
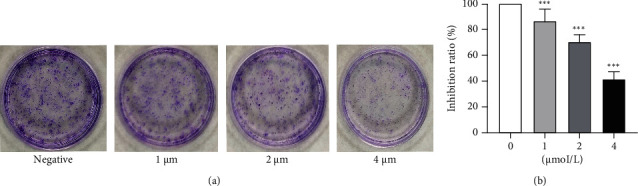
Anlotinib inhibited the proliferation of HSC-3 cells. HSC-3 cells were treated with different concentrations (0, 1, 2, and 4 *μ*M/L) of anlotinib for 10 days. (a) The effect of anlotinib on proliferation of HSC-3 cells. (b) Inhibition ratio of anlotinib on proliferation of HSC-3 cells. The results are from three independent experiments.

We further analyzed and confirmed the effects of anlotinib on the proliferation of HSC-3 cells, using a colony formation assay. By comparing the difference in the number of colonies between the groups containing different anlotinib concentrations and the control group, it was found that anlotinib significantly inhibited the number of colonies of HSC-3 cells, and the single-colony area was small, indicating that anlotinib can significantly inhibit the proliferation and colony formation of HSC-3 cells (*P* < 0.0001) (Figure 2).

### 3.3. Anlotinib Inhibited Oral Squamous Cell Carcinoma HSC-3 Cell Migration

**Figure 3:**
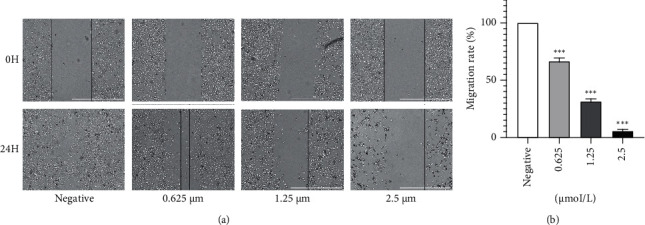
Effect of anlotinib on cell migration evaluated by wound healing assays in HSC-3 cells. The results are from three independent experiments ^∗∗∗^*P* < 0.001. (a) Effect of anlotinib on cell migration evaluated by wound healing assays in HSC-3 cells. (b) Effect of anlotinib on the migration of HSC-3 cells.

To determine whether anlotinib is related to the inhibition of HSC-3 cell migration, the inhibitory effect of anlotinib on the migration of HSC-3 cells was determined by a wound healing assay. By analyzing the results of cell scratch test, it was found that compared with the control group, when the drug concentrations in the administration group were 2.5, 1.25, and *μ*mol/L, the migration rates of HSC-3 cells were 2.98%, 26.54%, and 42.32%, respectively. The results showed that anlotinib had a good inhibitory effect on the migration of HSC-3 cells, and the difference was significant (*P* < 0.001) (Figure 3).

### 3.4. Anlotinib Inhibits HSC-3 Cell Migration by Suppressing the Activation of the Akt Pathway

**Figure 4:**
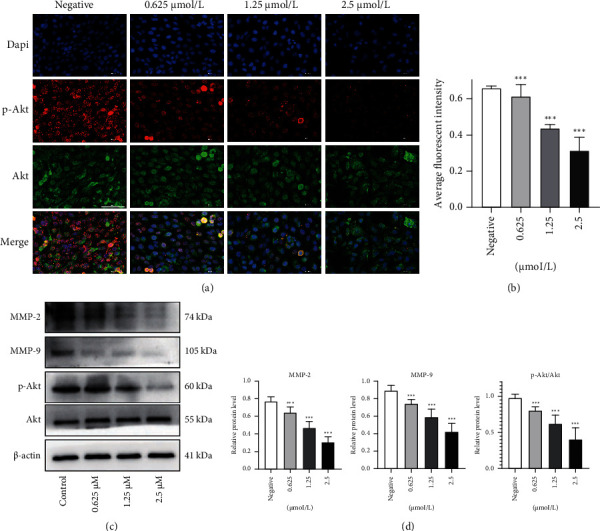
Anlotinib inhibits the migration of HSC-3 cells by suppressing the activation of the Akt pathway. (a) After HSC-3 cells were treated with different concentrations (0, 0.625, 1.25, and 2.5 *μ*M/L) of anlotinib for 24 h, expression of the p-Akt and Akt proteins was detected by immunofluorescence. (b) Quantitative immunofluorescence results of the p-Akt and Akt proteins. (c) HSC-3 cells were treated with anlotinib for 24 hours, and total MMP-2 and MMP-9, phosphorylated and total Akt and *β*-actin were detected by western blotting. (d) Ratio change of the migration markers MMP-2, MMP-9, phosphorylated, and total Akt. The data are expressed as the mean ± standard deviation of three repeats. ^∗∗∗^*P* < 0.001 compared with the control.

To further explore whether the inhibition of cell migration by anlotinib has relation to the Akt pathway, the role of p-Akt and Akt proteins in inhibiting migration was detected by immunofluorescence assay. The results showed that anlotinib downregulated the protein expression of p-Akt and Akt in HSC-3 cells during the inhibition of migration (Figures 4(a) and 4(b)). In addition, at migration-inhibitory concentrations, by comparison with the control group, western blotting results showed that the expression of p-Akt, Akt, MMP-2, and MMP-9 decreased gradually with the gradual increase in migration inhibition (Figures 4(c) and 4(d)). The above data showed that the utterance of p-Akt and Akt in HSC-3 cells decreased with increasing drug concentrations. Thus, anlotinib can significantly inhibit HSC-3 cell migration by targeting the Akt pathway.

### 3.5. The Effect of Anlotinib on Apoptosis in Oral Squamous Cell Carcinoma HSC-3 Cells

**Figure 5:**
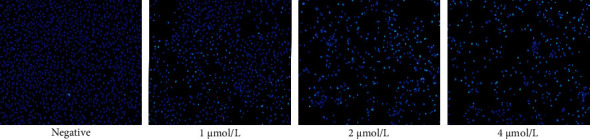
Anlotinib induced apoptosis in HSC-3 cells. After HSC-3 cells were treated with different concentrations (0, 1, 2, and 4 *μ*M/L) of anlotinib for 72 h, apoptosis was detected by Hoechst 33258 staining, which showed that apoptotic cells gradually increased from low to high drug concentrations.

To further verify whether anlotinib promotes apoptosis in oral squamous cell carcinoma HSC-3 cells, we attempted to determine the apoptosis of HSC-3 cells induced by anlotinib through activation of apoptotic bodies by Hoechst 33258 staining. The dye can penetrate the normal cell membrane slightly, and normal cells appear light blue. The cell membrane permeability of apoptotic cells was enhanced, and apoptotic cell nuclei are stained bright blue by the concentrated dye. Seventy-two hours after administration, compared with the blank control group, with increasing anlotinib concentrations, the normal HSC-3 cells decreased gradually, and the proportion of apoptotic cells increased significantly. We found that apoptosis increased with the drug concentration (Figure 5).

### 3.6. The Effect of Anlotinib on the RAS and PI3K/Akt Signalling Pathways in HSC-3 Cells

**Figure 6:**
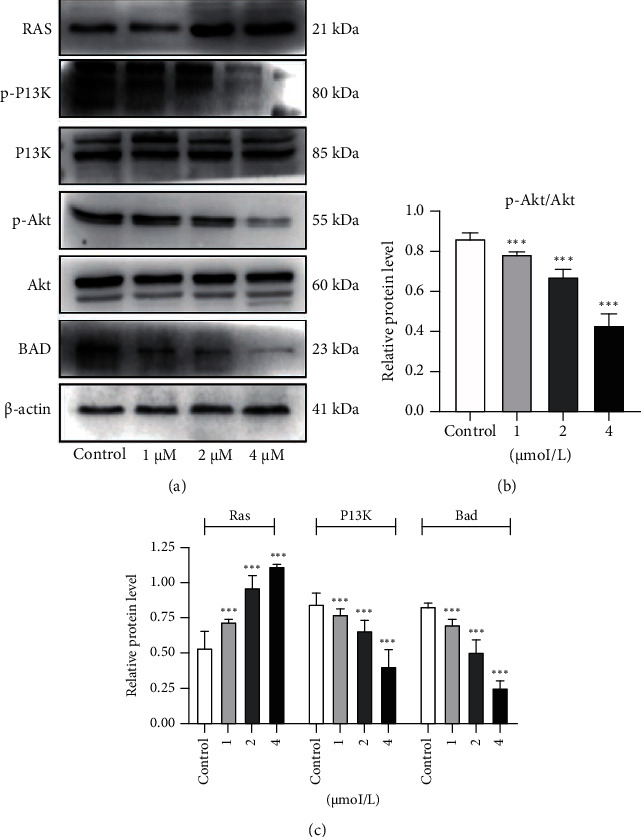
Effect of anlotinib on the RAS and PI3K/Akt signalling pathways in HSC-3 cells. HSC-3 cells were treated with anlotinib for 72 hours. Total RAS, phosphorylated and total Akt, and *β*-actin were detected by western blotting. (a) Molecular mechanism of anlotinib in HSC-3 cell apoptosis. (b) Changes in the p-Akt/Akt ratio in apoptosis. (c) Ratio changes of RAS, PI3K, and Bad proteins in apoptosis based on quantitative results of protein expression. The data are expressed as the mean ± standard deviation of three repeats. ^∗∗∗^*P* < 0.001 compared with the control.

Researches have shown that the RAS protein is closely related to tumour proliferation and apoptosis, and its downstream Akt pathway is closely related to cell migration. Therefore, we studied whether the promotion of apoptosis by anlotinib was related to the RAS protein and Akt pathway. Western blotting was used to detect the expression of HSC-3 cell signalling pathway-related proteins that promote apoptosis. At apoptotic concentrations, the intracellular RAS protein was activated (*P* < 0.0001), and the downstream PI3K protein expression level was downregulated (*P* < 0.001), thereby downregulating the expression level of the p-Akt protein. Therefore, the p-Akt/Akt ratio decreased (*P* < 0.0001). When the expression of the p-Akt protein was downregulated, expression of the Bad protein was downregulated (*P* < 0.0001), which inhibited expression of the Bad protein and promoted apoptosis. These results suggest that anlotinib activates the RAS protein and regulates the downstream PI3K/Akt pathway to promote apoptosis (Figure 6).

## 4. Discussion

Oral squamous cell carcinoma (OSCC) is the eighth most prevalent cancer in the world [16]. Heterogeneous tumors account for approximately 40% of head and neck squamous cell carcinomas (HNSCCs), including the tongue, upper and lower gums, oral base, and maxillary and buccal mucosa. OSCC is caused by the interaction of multiple factors, including tobacco, alcohol, and betel nut [16]. Since the early diagnosis rate is low, most patients are diagnosed in the late stage. Unfortunately, the five-year overall survival rate of OSCC is only 50% [17]. At present, the treatment of advanced OSCC mainly includes surgery, chemotherapy, radiotherapy, or a combination of these methods. Cisplatin, 5-fluorouracil, paclitaxel, and other chemotherapy drugs have become the first-line choice for OSCC patients [16]. However, most OSCC patients eventually develop drug resistance, leading to poor prognoses. Therefore, in order to improve the survival rate of OSCC patients, it is urgent to develop new effective alternative drugs.

Anlotinib, which is a novel oral multitarget receptor tyrosine kinase inhibitor, was first approved as the third-line treatment for refractory advanced non-small-cell lung cancer in May 2018 [18]. By regulating tumour cell proliferation, apoptosis, angiogenesis, migration, and invasion, anlotinib achieves encouraging efficacy and controllable and tolerable safety in a variety of malignant tumours, including medullary thyroid cancer, renal cell carcinoma, gastric cancer, and oesophageal squamous cell carcinoma [19]. However, the mechanism of anlotinib in OSCC remains to be further elucidated. Therefore, we conducted a series of studies. MTT results demonstrated that anlotinib was cytotoxic in HSC-3 cells, with IC50 values of 2.03 *μ*M for 24 h, 6.6207 *μ*mol/L for 48 h, and 1.001 *μ*mol/L for 72 h. In addition, wound healing assays and colony forming assays demonstrated that anlotinib suppressed the migration and proliferation of HSC-3 cells, and Hoechst 33258 staining showed that anlotinib promoted HSC-3 cell apoptosis. Consequently, the results suggested that anlotinib could significantly inhibit the cell growth of HSC-3 cells in vitro. Next, we further studied the inhibition of HSC-3 cell migration and proliferation by anlotinib and its mechanism of promoting apoptosis.

Akt is an important kinase activated by many cellular stimuli and has been recorded to be involved in various fundamental cellular physiological activities, including cell proliferation, apoptosis, autophagy, resistance, migration, and invasion [20]. Previous studies have shown that various intracellular and extracellular factors can upregulate the expression and migration of MMP-2 and MMP-9 by activating the Akt signalling pathway in OSCC [21]. To this end, we examined whether anlotinib inhibits HSC-3 cell migration through the Akt pathway. Western blotting experiments showed that the Akt pathway was gradually inhibited with increasing concentrations of anlotinib. In addition, MMP-2 and MMP-9 gradually decreased, indicating that anlotinib could inhibit the migration of HSC-3 cells by inhibiting the activation of the Akt pathway. However, studies of Akt pathway activators may be needed to further verify the effect of anlotinib on the migration of HSC-3 cells.

Cancer is mainly caused by normal cell gene mutations that lead to abnormal cell proliferation or apoptosis inhibition. Studies have shown that the RAS protein, as a cascade protein, plays a key role in the apoptosis of various tumour cells [22]. In our study, we found that anlotinib can significantly promote apoptosis in HSC-3 cells, and RAS protein expression gradually increased with increasing anlotinib concentrations. Furthermore, RAS can directly interact with the catalytic subunit of PI3KI, which catalyses the conversion of phosphatidylinositol 4,5-biphosphate (PIP2) to phosphatidylinositol 3,4,5-trisphosphate (PIP3), while PIP3 in turn activates downstream AKT [23]. Next, we evaluated the effect on the PI3K/Akt/Bad pathway. The results showed that the protein expression levels of p-PI3K/PI3K, p-Akt/Akt, and Bad were significantly inhibited by anlotinib, suggesting that anlotinib inhibited the activation of the Akt pathway. In summary, anlotinib may inhibit the activation of the Akt pathway by promoting the expression of the RAS protein, thereby promoting the apoptosis of HSC-3 cells.

Based on the current research results, we believe that anlotinib has a good inhibitory effect on oral squamous cell carcinoma HSC-3 cells in vitro. The above results help further facilitate in vivo experiments using anlotinib and the development of clinical medication and lay the foundation for the administration of combined medication and the improvement of clinical treatment. However, there are still many deficiencies. First, from the perspective of the mechanism, the specific target of anlotinib on RAS is not clear, and molecular docking may provide some clues. Hence, the targeted regulation of anlotinib can be further studied. Second, the persuasiveness of a single HSC-3 cell line may be weak; thus, further in vivo research is necessary. Third, the administration and toxicity accumulation of anlotinib in the treatment of OSCC also need further analysis. Studies have shown that drug therapy has multiple effects on such complex systems [24–26]. Therefore, in the study of the new drug anlotinib, its role in different systems can also be further examined.

## 5. Conclusion

In conclusion, this study demonstrated that anlotinib inhibits the activation of the PI3K/Akt pathway through the RAS protein, thereby significantly inhibiting the proliferation and migration of HSC-3 cells and promoting apoptosis, presenting a promising potential therapy for patients with OSCC.

## Data Availability

The data used to support the findings of this study are included within the article.
